# Leupaxin is expressed in mammary carcinoma and acts as a transcriptional activator of the estrogen receptor α

**DOI:** 10.3892/ijo.2015.2988

**Published:** 2015-05-06

**Authors:** SILKE KAULFUSS, ANNA-MARIA HERR, ANJA BÜCHNER, BERNHARD HEMMERLEIN, ANDREAS R. GÜNTHERT, PETER BURFEIND

**Affiliations:** 1 Institute of Human Genetics, University Medical Center Göttingen, Germany; 2 Department of Pathology, University Medical Center Göttingen, Germany; 3 Department of Gynaecology and Obstetrics, University Medical Center Göttingen, Germany; 4 Department of Pathology, HELIOS Klinik, Krefeld, Germany; 5 Department of Gynaecology and Obstetrics, Cantonal Hospital of Lucerne, Lucerne, Switzerland

**Keywords:** leupaxin, breast cancer, nuclear export signal, estrogen receptors, invasion

## Abstract

Leupaxin belongs to the group of paxillin proteins and was reported to play a major role in the invasion and migration of prostate cancer cells. In the present study we were able to show by using a cDNA cancer profiling array that leupaxin is upregulated in breast and endometrial cancer, whereas downregulation of leupaxin was observed in lung cancer. In addition, immunohistochemical studies using a leupaxin-specific antibody on human breast cancer specimens (n=127) revealed that leupaxin is expressed mainly in invasive ductal carcinomas and ductal carcinoma *in situ* (40 and 49% respectively), and only in a minority of lobular mammary carcinomas. To further investigate the role of leupaxin in the progression of breast cancer the expression of leupaxin was analysed in six breast cancer cell lines. The estrogen receptor α (ERα)-positive HCC70 and the ERα-negative MDA-MB-231 cells showed leupaxin expression on the RNA and protein level. Leupaxin localizes in these mammary carcinoma cells at focal adhesion sites and shuttles between membrane and nucleus via its LD4 motif as major nuclear export signal. Interaction partners of leupaxin in the nucleus represent the estrogen receptors ERα and ERβ. Both ERα and ERβ bind to the LIM domains of leupaxin via their AF-1/DNA binding domains. Furthermore, leupaxin is able to induce transcriptional activity of ERα independent of the presence of estradiol. The specific downregulation of leupaxin expression using siRNAs in mammary carcinoma cells resulted in reduced migratory capability and diminished invasiveness whereas no effect on proliferation was observed. Collectively, these results show that leupaxin has particular influence on the progression and invasion of breast cancer cells and may therefore represent an interesting candidate protein for diagnosis and therapeutic interventions.

## Introduction

Breast cancer is the most common solid cancer in women around the world and the leading cause of cancer-related deaths. After initial surgery adjuvant treatment strategies include cytotoxic chemotherapy, radiation therapy, and anti-hormonal therapy (1). Advances in earlier diagnosis and therapy have significantly improved outcomes. However, recurrent metastatic breast cancer is still incurable and only 3% of patients with metastatic disease achieve a complete response for >5 years after combination chemotherapy (2,3); the median survival time after therapy is ~2 years.

Endogenous estrogens are thought to play a major role in the development of breast cancer, and estrogen receptors (ER) are targets of hormonal therapy. These nuclear receptors are ligand-dependent transcription factors that mediate the biological effects of (anti-) estrogens. There are two types of specific receptors: ERα and ERβ, which differ in their responses to agonists and antagonist due to differences in their C-terminal ligand binding domains (LBD) (4). The receptors show different expression patterns in breast cancer tissues and seem to have opposing roles in the proliferation of breast cancer cells. ERα-positive tumours are related to a good prognosis, and it is suggested that ERβ expression declines during breast tumour genesis (5). Despite high ERα levels in some primary tumours and in all patients with metastatic disease resistance to endocrine therapies arise. The potential mechanisms for either intrinsic or acquired endocrine resistance are still poorly comprehended, but they include cross-talk between the ER pathway and other growth factor and kinase networks as well as ER-co-regulatory proteins (6). Increased expression of co-activator proteins that mediate ER activity or downregulation of co-repressor activity reducing the inhibitory potential of tamoxifen are possible molecular mechanisms for resistance and the progression of confined breast cancer to invasive disease (7–9).

The paxillin protein family, which comprises paxillin, transforming growth factor β1 induced transcript 1 (TGFB1I1 or Hic-5) and leupaxin, is involved in the majority of the steps during cell migration and invasion as part of the focal adhesion complexes. It is also known, that all of them can interact with different steroid hormone receptors and induce their transcriptional activity in the nucleus (10,11), thus serving as candidate proteins involved in the response to hormonal therapy. All members of the paxillin protein family contain two different protein-protein interaction domains, namely LD motifs and LIM domains. LD motifs contain two invariant amino acids, leucine and aspartate (LD). LIM domains are composed of two zincfinger domains and were identified in the transcription factors LIN -11, ISL-1 and MEC-3 (LIM). Due to this protein structure paxillin proteins represent adaptor platforms in transmitting signals from outside the cell to affect transcriptional regulation in the nucleus. Originally identified in hematopoietic cells, leupaxin was found to be expressed in a series of other tissues, e.g., smooth muscle cells and prostate cancer cells (12,13). Recently, it was shown that leupaxin expression in human prostate cancer correlates with tumour stage and that leupaxin downregulation in prostate cancer cells results in decreased migratory ability and invasiveness. Furthermore, we showed that leupaxin functioned as a co-activator of the androgen receptor (13).

In the present study, we further elucidated the role of leupaxin in different human cancers. We showed that leupaxin is expressed in breast and endometrial carcinomas. Downregulation of leupaxin expression in breast cancer cells decreases migration and invasion. In addition, leupaxin shuttles between focal adhesion sites and the nucleus and interacts with both ERs via their N-terminal parts. This interaction results in the transcriptional activation of ERα in the presence and absence of ER ligands. Taken together, our results underline the role of leupaxin as an important factor in the progression of breast cancers and give a further basis to investigate the potential of leupaxin as a target for the development of novel therapeutic strategies.

## Materials and methods

### Cancer profiling array

The Cancer Profiling Array I (BD Biosciences Clontech, Heidelberg, Germany) was hybridized according to the manufacturer’s instructions with a [^32^P]-labelled leupaxin cDNA (nucleotide position 437-1748; NM_004811) probe, using the Rediprime II labelling kit (GE Healthcare GmbH, Freiburg, Germany). After overnight hybridization and a high-stringency wash, the array was scanned and analysed with a Molecular Imager FX and Quantity One Software (Bio-Rad, Hercules, CA, USA).

### Patient material and immunohistochemistry

Ethical approval was obtained from the ethics committee of the University of Göttingen for the use of human material in the present study. Immunohistochemistry was performed as described previously (13). The mouse monoclonal anti-leupaxin (clone 283 G) antibody was kindly provided by Eli Lilly & Co. (Indianapolis, IN, USA). For negative controls, blocking solution was used in place of the primary antibody.

### Semiquantitative analysis of leupaxin immunoreactivity and statistical analysis

For quantification of the leupaxin immune signals in tissue sections an additive immunoreactive score (IRS = SI + CN) was applied comprising the average signal intensity (SI) and the number of positive tumour cells (CN) (13). For comparative analyses of leupaxin immunoreactivity with clinicopathologic features the χ^2^ test and Fisher’s exact test were applied.

### Cell culture and transient transfection

MDA-MB-231, MDA-MB-453, HCC70, ZR-77-1, MCF-7, T-47D and NIH/3T3 cells were purchased from ATCC and grown in DMEM medium (PAN-Systems, Nuremberg, Germany) containing 10% FCS and 1.2% antibiotics. For MCF-7 and T-47D 1% non-essential amino acids was added. Transient transfection experiments were performed using FuGENE (Roche Diagnostics GmbH, Mannheim, Germany) according to the manufacturer’s instructions.

### Northern blot analysis

Northern blot analysis was performed as described previously (14). Total RNA from breast cancer cell lines was isolated using RNeasy MINI (Qiagen, Hilden, Germany). Total RNA (5 μg) was separated and hybridized with the leupaxin probe mentioned above.

### Western blot analysis and immunoprecipitation

Whole-cell lysates from parental and transfected breast cancer cells were prepared using lysis buffer and subjected to western blot analysis as described previously (13). The following primary antibodies were used: mouse monoclonal anti-leupaxin 283 C (kindly provided by Eli Lilly & Co.), mouse monoclonal anti-α-tubulin (Sigma, Taufkirchen, Germany), rabbit polyclonal anti-ERα (MC-20, Santa Cruz Biotechnology, Dallas, TX, USA) and rabbit polyclonal anti-ERβ (Cell Signaling Technology, Danvers, MA, USA). For coimmunoprecipitation assay cells were lysed with IP buffer (0.05 M Tris pH 7.5, 0.15 M NaCl, 0.5% deoxycholic acid, 1% IGPAL) in the presence of proteinase inhibitor cocktail (Roche) and 3 mg protein was used for immunoprecipitation with 1 μg ERα and ERβ antibodies, respectively. After incubation overnight at 4°C, 50 μl protein A/G sepharose (Santa Cruz Biotechnology) was added and incubated for 2 h. Protein complexes were isolated by centrifugation and three washes with IP buffer and final elution with 2X SDS sample buffer (Cell Signaling Technology). Subsequently, western blotting was performed. Immunoprecipitation was performed in three independent experiments.

### Immunocytochemistry

Cells were plated on culture slides coated with 10 μg/ml fibronectin (Sigma) under normal culture conditions. The cells were then fixed with 4% formaldehyde in PBS, permeabilised with 0.1% Triton X-100 in PBS and blocked with 3% BSA in PBS for 30 min at room temperature. After incubation with primary antibodies (10 μg/ml mouse monoclonal anti-leupaxin) overnight at 4°C, cells were washed with PBS and incubated for 2 h at RT with secondary antibodies (1:500 sheep anti-mouse-IgG-Cy3, Sigma). Subsequently, cells were washed in PBS, stained with FITC-phalloidin (Sigma) for 30 min and mounted using Vectashield/DAPI. Images were acquired using the Olympus FluoView1000 confocal scanning microscope and FluoView software (Olympus Deutschland GmbH, Hamburg, Germany).

### Direct yeast two-hybrid experiments

The yeast two-hybrid experiments were carried out by using the Matchmaker GAL4 two-hybrid system (Clontech Laboratories, Inc., Saint-Germain-en-Laye, France). All procedures were performed according to the manufacturer’s protocols. The plasmids pGADT7-LPXN, pGADT7-LPXN-LD and pGADT7-LPXN-LIM were described previously (13). The open reading frames of ERα (*Eco*RI) and ERβ (*Nco*I/*Sal*I) were cloned into the pGBKT7 vector. Plasmids were co-transformed into the yeast host strain AH109. Co-transformants were selected in the presence or absence of 100 nM estradiol (Sigma) on minimal synthetic dropout (SD) medium lacking the amino acids leucine, tryptophan, histidine and adenine (SD-LTHA) containing 80 mg/l X-Gal (ICN).

### ERα transactivation assay

pcDNA-ERα was cloned by amplification of the ERα open reading frame (361-2148, NM_000125) and cloning into the *Eco*RI restriction site of pcDNAmyc/HisA vector (Life Technologies, Darmstadt, Germany). Reporter gene assays were performed as described previously (13) using charcoal-stripped FCS and phenol-red free medium. Cells were transfected with the following expression vector cocktail after 24 h: 0.05 μg pCMV-β-Gal, 0.05–0.4 μg GFP-LPXN (as indicated) and with 0.2 μg Vit-ERE-Luc and with 0.2 μg pcDNA-ERα vector. Thirty-six hours after transfection cell lysates were prepared and luciferase activity was measured in a microplate luminometer (LB953, Berthold) by injecting 100 μl of a luciferin solution (P.J.K. GmbH, Kleinblittersdorf, Germany) per well. The luciferase activity was normalized against the β-galactosidase activity, which was measured by using the Galacto-Light™ kit (BD Bioscience) according to the manufacturer’s protocol.

### RNA interference

Transfection of cells was accomplished using Oligofectamine reagent (Life Technologies) according to the manufacturer’s instructions with leupaxin gene-specific siRNA duplexes as described previously (13). Control cells were transfected with siRNA duplex oligonucleotides against the firefly luciferase gene (15). At different time-points after transfection (24, 48 and 72 h) cells were collected and used in the following experiments.

### Real-time RT-PCR analysis

Real-time RT-PCR analysis was performed as described previously (13). Primers used for quantitative RT-PCR were: PBGD-For-QGCAATGCGGCTGCAACGGCGGAAG; PBGD-Rev-QCCTGTGGTGGACATAGCAATGATT; TBP-For-QAGCCTGCCACCTTACGCTCAGTBP-Rev-QTGCTGCCTTTGTTGCTCTTCCA; leupaxin-Q4-Fw AGTTCCTTTGCGGTCCTCTTCTTC; leupaxin-Q4b-Rev GTCTCCTTTCTGGAATGCTGATCC.

### Invasion and migration assay

Cell invasion was determined in BioCoat Matrigel Invasion Chambers (BD Biosciences) as described previously (13,15). siRNA transfected cells (2.5×10^4^ cells, respectively) were incubated in invasion chambers for 22 h at 37°C. To estimate directional migration the transfected MDA-MB-231 cells were plated on Millicell^®^ Cell Culture Inserts (Merck Millipore, Darmstadt, Germany) with 6×10^4^ cells/well and incubated for 24 h. Invaded and migrated cells were stained with haematoxylin and eosin and counted from five randomly chosen fields under a BX60 microscope using the analySIS software (Olympus). Data are expressed as the percentage of cells with reduced leupaxin expression in comparison to control transfected cells.

### Statistical analyses

If not otherwise stated, experiments were performed at least three independent times (biological replicates). For statistical analysis Student’s t-test was applied. ^*^p≤0.05, significant; ^**^p≤0.01, very significant; ^***^p≤0.001, extremely significant; NS, not significant.

## Results

### Leupaxin is expressed in different types of cancer

**Figure 1:**
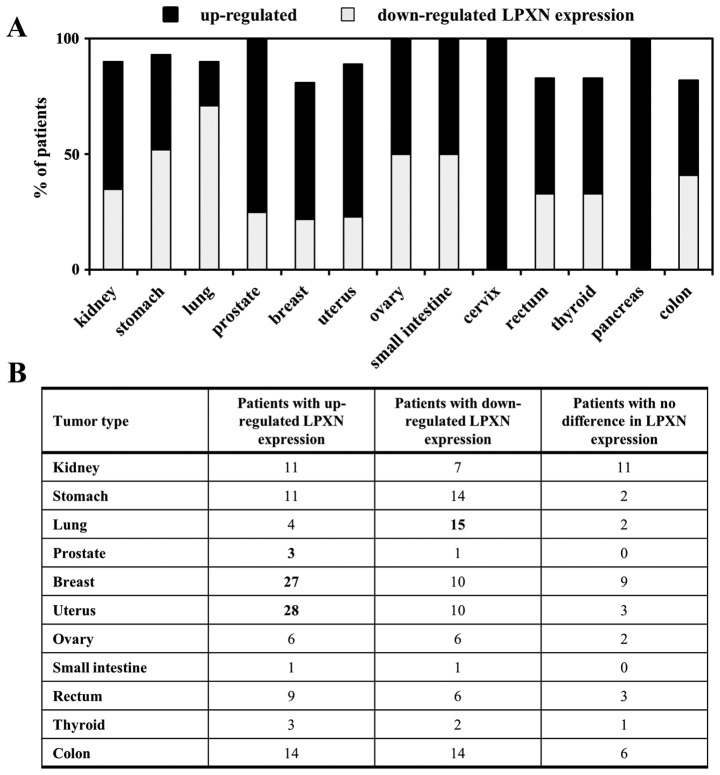
Leupaxin expression in a variety of cancer types. The cDNA Cancer profiling array was hybridized with a leupaxin-specific probe to analyse for leupaxin expression in matched normal and tumour tissues. (A) The expression of leupaxin in normal and tumour tissue was quantified and the percentage of patients with up or downregulation of leupaxin expression in the tumour parts was blotted. A clear upregulation of leupaxin expression was observed in prostate, breast and uterus. For cervix and pancreas only one patient was analysed. (B) Summary of the results obtained from the Cancer profiling array.

Recent studies indicated that the expression of leupaxin is not limited to cells of hematopoietic origin. Therefore, a cancer profiling array analysis with a human specific leupaxin probe was performed to investigate the expression profile of leupaxin in normal and matched tumour tissue samples. As shown in Fig. 1 most breast and endometrial cancer patients displayed upregulation of leupaxin expression in the tumour as compared to the normal tissue, whereas in lung cancer patients downregulation of leupaxin expression was clearly observed. It is noteworthy that upregulation of leupaxin expression was detectable in three out of four prostate cancer tissues confirming previous results of our group (13). Other cancer types showed an equal distribution of patients with up or downregulated leupaxin expression, e.g., ovarian and colon cancer, and were therefore considered to be not relevant in this study.

### Leupaxin is expressed in mammary cancer tissue

**Figure 2:**
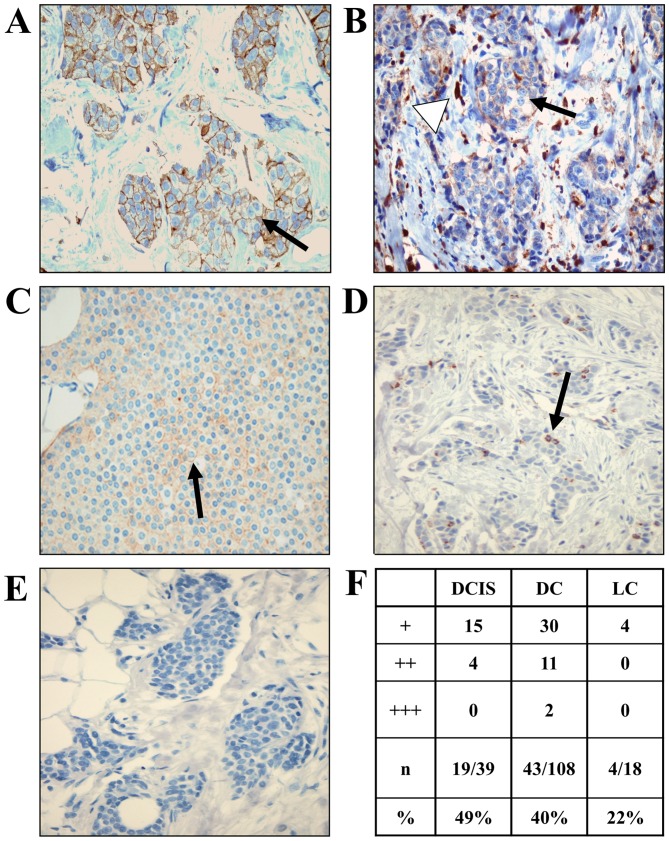
Immunohistochemical analysis of cellular leupaxin expression in human breast cancers. Sections of 127 breast cancer specimens were stained with a leupaxin specific antibody and counterstained with hemalum. Tumours were classified into ductal carcinoma *in situ* (DCIS), invasive ductal (DC) or invasive lobular (LC) carcinomas. (A and B) Invasive ductal carcinoma with high (A) and low (B) expression of leupaxin (arrow). Infiltrating lymphocytes (arrowhead) showed strong leupaxin expression and served as internal positive control. (C) Cytoplasmic and membrane staining of leupaxin in a ductal invasive carcinoma (arrow). (D) A few cases showed a perinuclear signal of leupaxin expression in tumour tissues (arrow). (E) Negative controls to show specificity of the leupaxin staining were conducted using non-immune serum. (F) Staining was scored by considering signal intensity and proportion of positive tumour cells (see Materials and methods). Forty-nine percent of DCIS tumours, 40% of DC and 22% of LC showed overexpression of leupaxin. All images were obtained with ×400 magnification.

To evaluate the relevance of leupaxin expression in breast cancer, 127 tissue sections from breast cancer patients were stained with a leupaxin specific antibody and classified into low, medium and high depending on the percentage of positive cancer cells and the according leupaxin expression level. Mammary carcinomas were classified in ductal carcinoma *in situ* (DCIS), invasive ductal (DC) or invasive lobular (LC) carcinomas (Fig. 2A–E). Different cancer types in one sample were individually evaluated. As seen in Fig. 2F, 49% of ductal carcinoma *in situ* and 40% of invasive ductal carcinomas displayed leupaxin expression. Only 22% of LC carcinomas showed staining for leupaxin. There was no significant correlation between the expression level of leupaxin and the tumour stage or hormone receptor status of ERα and progesterone receptor (PR) as well as HER2, respectively. However, we observed higher staining scores (++ and +++) only in more advanced breast cancers (DC).

### Expression of leupaxin in mammary carcinoma cell lines

**Figure 3:**
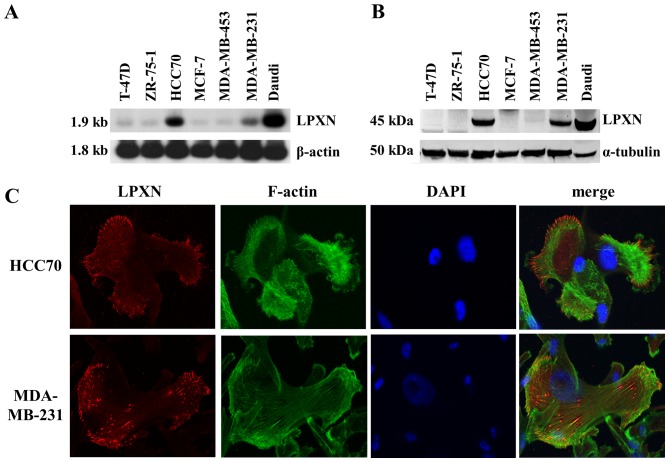
Expression of leupaxin in breast cancer cell lines. (A and B) Northern (A) and western blot analysis (B) of leupaxin expression in the depicted breast cancer cell lines show highest expression in HCC70 and MDA-MB-231 cell lines. (C) HCC70 and MDA-MB-231 cells were stained with a leupaxin specific antibody (red). The F-actin and nuclei were stained using FITC-conjugated phalloidin and DAPI, respectively. Leupaxin is mainly localized at focal adhesion sites. Images were obtained using a confocal laser microscope with ×600 magnification.

**Figure 4:**
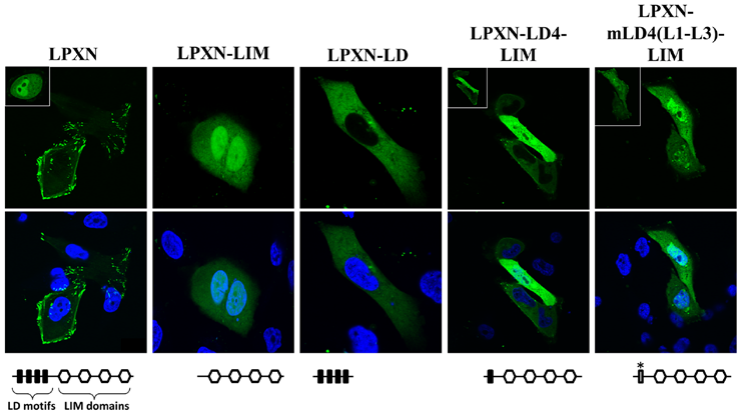
Leupaxin shuttles between cytoplasm and nucleus. MDA-MB-231 cells were transfected with different leupaxin-EGFP fusion constructs, fixed after 36 h and analysed using confocal microscopy. Images were obtained with ×600 magnification. Drawings of the composition of the used leupaxin-EGFP fusion constructs are depicted. Full-length leupaxin (LPXN) contains all four LD motifs and LIM domains and preferentially locates at focal adhesion sites. A few cells (~1%) showed nuclear accumulation of leupaxin (inlet). LPXN-LIM and LPXN-LD comprise only the LIM domains and LD motifs, respectively. LPXN-LIM accumulates in the nucleus, LPXN-LD is fully exported out of the nucleus. Whereas LPXN-LD4-LIM, containing the four LIM domains plus the last LD motif, was localized in 53% of cells in the nucleus, LPXN-mLD4(L1–L3)-LIM, which consists of a mutated LD4 motif and the LIM domains, showed in nearly 100% of transfected cells a nuclear accumulation. Localization of LPXN-LD4-LIM and LPXN-mLD4(L1–L3)-LIM to the focal adhesion sites is not impaired (inlets).

The expression of leupaxin was evaluated in seven established breast cancer cell lines. Northern (Fig. 3A) and western blot (Fig. 3B) analyses demonstrated, that leupaxin is highly expressed in the ER-negative MDA-MB-231 and in the ER-positive HCC70 cell lines, whereas no expression was detectable on the protein level independent of ER status or invasive behaviour in the other analysed cell lines. Subcellularly, leupaxin localized to the focal adhesion sites in MDA-MB-231 and HCC70 cells (Fig. 3C). Furthermore, MDA-MB-231 cells were transfected with EGFP-constructs coding for different EGFP-LPXN fusion proteins as indicated in Fig. 4. The full-length EGFP-LPXN fusion protein is located in the focal adhesion sites and in a small proportion of the nucleus. EGFP-LPXN-LIM, which contains only the LIM domains, localizes to the nucleus in 100% of the cells. If the LD4 motif is present in the fusion protein (EGFP-LPXN-LD4-LIM) only 53% of cells show a nuclear accumulation, but, if the LD4 motif is mutated, nuclear distribution is detectable in all transfected cells (EGFP-LPXN-mLD4(L1-L3)-LIM) (Fig. 4). These studies clearly demonstrate that leupaxin shuttles to the nucleus in breast cancer cells and that mainly the LD4 is responsible for the nuclear export of leupaxin.

### Leupaxin interacts with the estrogen receptors α and β

**Figure 5:**
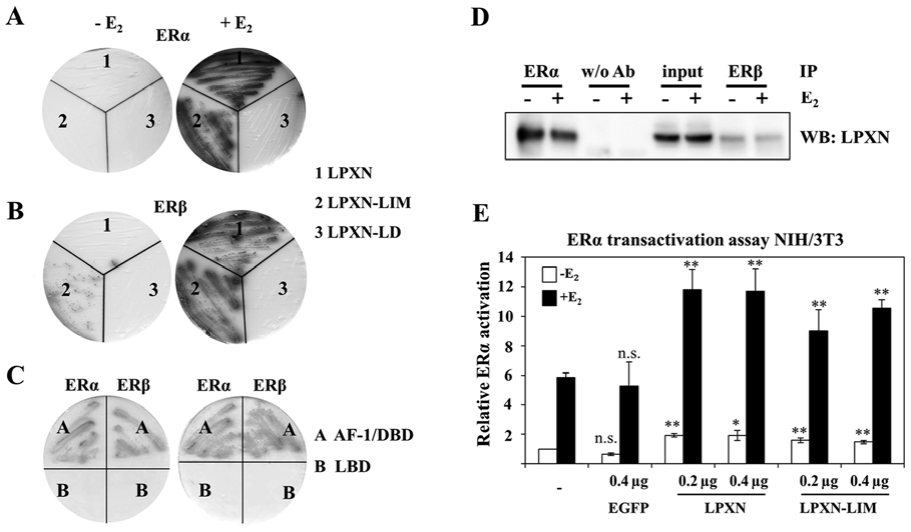
Leupaxin interacts with the estrogen receptors α and β. (A and B) Direct yeast-two-hybrid experiments were performed using full-length ERα (A) or full-length ERβ (B) and leupaxin (1, full length), LPXN-LIM (2, containing all four LIM domains) and LPXN-LD (3, containing all four LD motifs), respectively. Transformed yeasts were plated on drop-out plates (-LTHA) in the absence or presence of estrogen (E_2_). Growth and blue staining of yeast show interaction of ERα and ERβ with the indicated leupaxin protein, respectively. (C) Interaction of leupaxin with ERs was further analysed with different ER proteins. The N-terminal part of ERα and ERβ containing AF-1 and the DNA binding domain (A) interacts with full-length leupaxin in the absence and presence of E_2_ whereas no interaction was observed using leupaxin and the ligand binding domain (LBD) (B) of ERα and ERβ, respectively. (D) Co-immunoprecipitation experiments verified interaction of leupaxin with ERα and ERβ in HCC70 cells. In contrast to the yeast experiment an involvement of estrogen was not observed. (E) Leupaxin enhances ERα transcriptional activity. NIH3T3 cells were transfected with plasmids pCMV-β-Gal, Vit-ERE-luc, pCDNA-ERα and the indicated amount (0.2 or 0.4 μg DNA) of EGFP and EGFP-LPXN constructs in the absence or presence of E_2_. Luciferase activity was measured 36 h after transfection and normalized against β-galactosidase activity. Three independent experiments were performed. For statistical analysis Student’s t-test was applied to compare with parental cells. ^*^p≤0.05; ^**^p≤0.01; ^***^p≤0.001; NS, not significant.

As it was shown that leupaxin interacts and activates the androgen receptor in prostate cancer cells, a putative interaction between leupaxin and the ERs α and β in breast cancer cells was investigated using direct yeast-two-hybrid experiments. Competent yeast cells of the strain AH109 were transformed with plasmids coding for the full-length leupaxin (LPXN), LPXN-LIM (containing only the LIM domains) or with LPXN-LD (containing only the LD motifs) fused to the GAL4 activation domain (AD) together with plasmids coding for the ERα and ERβ fused to the DNA binding domain of the GAL4 transcription factor. Transformed yeast cells were plated with or without estradiol on high stringency drop-out plates containing α-Gal. An interaction of the analysed proteins is reasoned upon growth of the yeasts and blue colour development. As shown in Fig. 5 leupaxin interacts with the ERα only in the presence of estradiol and via its LIM domains. In contrast, leupaxin binds to the ERβ via its LIM domains in the absence and presence of estradiol (Fig. 5). However, ERβ showed interaction with the full-length leupaxin (LPXN) only in the presence of estradiol.

To verify the interaction of leupaxin and ERα and ERβ, respectively, coimmunoprecipitation experiments were performed. HCC70 cells were incubated in the presence or absence of estradiol and total protein was isolated. For immunoprecipitation an ERα and an ERβ-specific antibody, respectively, and for western blotting a leupaxin specific antibody were applied. An interaction of leupaxin and the ERα and ERβ was observed in the presence and absence of estradiol, contrary to the yeast-two-hybrid results. No interaction was detectable in the control setting (without primary antibody).

### Leupaxin enhances the transcriptional function of the ERα

To analyse the biological relevance of leupaxin-ERα interactions a transactivation assay using pVit-ERE-Luc as reporter construct was performed. NIH/3T3 cells were chosen for the assay to rule out any influence of endogenous active estrogen receptors. Cells were transfected with pVit-ERE-Luc along with the plasmids EGFP-LPXN or EGFP-LPXN-LIM, respectively, and pcDNA-ERα. Measurement of luciferase activity clearly demonstrates that leupaxin increases the transcriptional activity of the ERα mainly in the presence of estradiol. There is a slight but significant increase of transcriptional activity visible without estradiol when using the full-length leupaxin construct supporting the co-immunoprecipitation studies.

### Leupaxin influences migration and invasion of breast cancer cells

**Figure 6:**
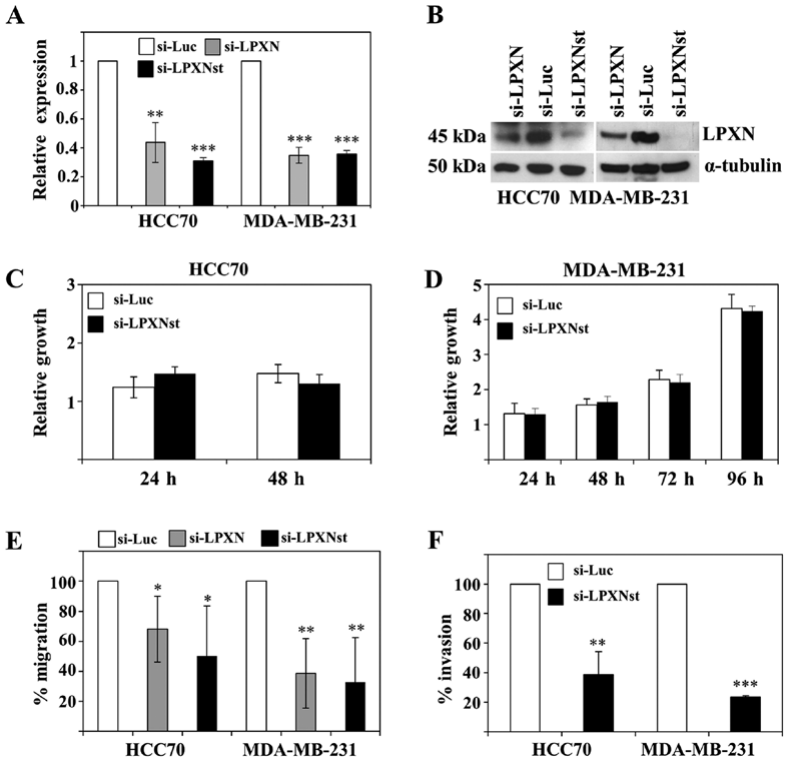
Downregulation of leupaxin expression decreases migration and invasion of breast cancer cells. HCC70 and MDA-MB-231 cells were transfected with leupaxin specific siRNAs si-LPXN and si-LPXNst. As control siRNA against the luciferase gene was used. (A and B) RNA and protein was isolated after 72 h and subjected to quantitative RT-PCR (A) and western blot analysis (B), respectively. Strongest downregulation of leupaxin expression was revealed with si-LPXNst. HCC70 (C) and MDA-MB-231 (D) cells were analysed for cell proliferation using MTT assay after the indicated time-points. No influence of leupaxin on cell proliferation was observed. (E and F) Reduction of leupaxin expression results in ≤70% reduced migration in a transwell migration assay (E) and in ≤70% reduction of invasion in a Boyden chamber assay (F) in HCC70 and MDA-MB-231 cells. (A–F) Three independent experiments were performed. For statistical analysis Student’s t-test was applied to compare with control transfected (si-Luc) cells. ^*^p≤0.05; ^**^p≤0.01; ^***^p≤0.001; NS, not significant.

To analyse the function of leupaxin in breast cancer cells, MDA-MB-231 and HCC70 cells were transfected with two leupaxin specific siRNAs (si-LPXN and si-LPXNst) and as a control with siRNA against the firefly luciferase gene (si-Luc). Downregulation of leupaxin expression was confirmed on the RNA and on the protein level by using quantitative RT-PCR and western blotting, respectively (Fig. 6A and B), showing highest efficiency for si-LPXNst.

As already shown for prostate cancer cells leupaxin has no influence on the proliferation of HCC70 and MDA-MB-231 cells (Fig. 6C and D). Leupaxin knockdown cells were subsequently analysed for their migratory capability. HCC70 and MDA-MB-231 cells with downregulated leupaxin expression show an ≤70% diminished migratory ability than control transfected cells (Fig. 6E). Furthermore, a Matrigel invasion assay revealed a 63 and 77% reduced invasiveness of HCC70 and MDA-MB-231 cells, respectively, with reduced leupaxin expression (Fig. 6F).

## Discussion

Breast cancer development and progression is critically influenced by ERs. Especially ERα, which is expressed in ~75% of breast cancers, is the most important target in endocrine treatment strategies. Serial treatment at tumour progression with different endocrine agents is the standard therapy strategy, often resulting in a long period of disease control (6). However, most patients with advanced breast cancer will develop resistance to endocrine therapy and different mechanisms of resistance have been described (6,16). In addition to modifications of the ERα itself, crosstalk with growth factor receptor signalling pathways and the deregulation of co-factors involved in the proper ERα signalling have been described (7–9). In the present study, we provide evidence that the focal adhesion protein leupaxin is involved in the regulation of ER action as a co-factor and that it is expressed in breast cancers, but not in normal breast epithelial cells. We obtained the first hints of an involvement of leupaxin in breast cancer from an array study containing patient matched tumour and normal tissues of a variety of cancer types. Leupaxin was found to be overexpressed in >50% of tumours in the breast and uterus, whereas in lung cancer 71% of tumours showed downregulation of leupaxin expression. Further analysis of leupaxin expression in 127 breast cancer specimens showed expression of leupaxin in 49, 40 and 22% of DCIS, DC and LC, respectively. However, there was no significant correlation of leupaxin expression with the nodal, HER2 or ER/PR status. This result was also reflected in the analysis of leupaxin expression in breast cancer cell lines with different receptor status (Fig. 3). However, more pronounced staining of leupaxin was observed mainly in DCs, which represents the more advanced breast cancer stage. Paxillin was also previously studied in breast cancers but with different outcomes. Whereas no correlation of paxillin expression with ER, PR and HER2 status in imprint smears of aggressive breast cancers was found (17), Short *et al* provided evidence that paxillin is overexpressed in 28% of breast cancers, and that this correlates with HER2 status (18). *In vitro* studies concerning paxillin function during breast cancer lung metastasis identified paxillin as a key regulator of 3D adhesion assembly, stabilization and disassembly (19). This afore-mentioned study also showed a contribution of Hic-5 (TGFB1I1) in the metastatic process, but up to date, to our knowledge, there are no data of Hic-5 expression in human breast cancer available.

To further evaluate if leupaxin plays an important role also during breast cancer progression we used HCC70 and MDA-MB-231 showing highest leupaxin expression for further analyses. Neither paxillin nor Hic-5 interaction with ERs was described (10,11). In contrast, leupaxin interacts via its LIM domains with the N-terminal part of the ERs [comprising activation function-1 (AF-1) and DNA binding domain]. However, whereas the interaction of leupaxin with ERα in our yeast-two-hybrid experiments was highly estrogen-dependent, co-immunoprecipitation studies could not verify this observation. Of note, the observed estrogen dependence of ERα/leupaxin interaction was abrogated when we performed the yeast-two-hybrid experiments with only the N-terminal part of the ERα which does not contain the estrogen binding site. In addition, reporter gene studies in NIH3T3 cells, which do not express ERα, showed that in the presence of estrogens the activation of ERα through leupaxin is enhanced, but the overexpression of leupaxin in the absence of estrogens is also sufficient to increase ERα transcriptional activity (Fig. 5E). These results lead to the idea that the interaction of leupaxin with the ERα in the cellular context might be regulated also through other factors thereby determining the estrogen dependence of the interaction.

To influence transcriptional activity of steroid hormone receptors paxillin proteins have to shuttle to the nucleus. The precise import mechanisms are not fully understood, but it is known that paxillin, Hic-5 and leupaxin contain a nuclear export signal (NES) within the N-terminal LD motifs (13,20,21). Staining of HCC70 and MDA-MB-231 cells with a leupaxin specific antibody revealed mainly localization of leupaxin at focal adhesion sites (Fig. 3C). A few cells with overexpression of leupaxin as a EGFP fusion protein showed strong accumulation of leupaxin in the nucleus as well (Fig. 4). From prostate cancer cells it is known that the leupaxin-LD4 motif is most important for nuclear export of leupaxin. Mutation of important amino acids within this motif led to the accumulation of leupaxin in the nucleus in MDA-MB-231 cells demonstrating that leupaxin also shuttles between cytoplasm and nucleus in breast cancer cells.

To further explore the involvement of leupaxin in breast cancer we knocked down leupaxin expression using previously established siRNAs (13). We showed that reduction of leupaxin expression did not result in diminished cell proliferation. Instead, a clear reduction of migration and invasion was observed. Of note, primary cancer-derived cell lines MDA-MB-231 and HCC70 showed highest expression of leupaxin. All other cell lines derived from breast cancer metastases showed low or even no expression of leupaxin. This fact points to the hypothesis, that leupaxin-mediated invasiveness is more important for the extravasation, but not for the intravasation of cells at metastatic sites. Expression analyses for leupaxin in metastases of breast cancers may strengthen this hypothesis. This second function of leupaxin is independent of its ERα co-activation function as we observed the effects in ERα-negative MDA-MB-231 cells as well.

In conclusion, we showed in the present study that leupaxin is overexpressed in human breast cancers. Supported by the results of the *in vitro* studies we postulate leupaxin to be an important player during breast cancer progression. Therefore, leupaxin and its involved genes and pathways could serve as potential targets in the development of new therapeutic strategies for breast cancer.
